# Impact of Type 1 Diabetes on Exercise Capacity and the Maximum Level of Peripheral Fatigue Tolerated

**DOI:** 10.3390/jcm15031252

**Published:** 2026-02-04

**Authors:** Nadia Fekih, Amal Machfer, Halil İbrahim Ceylan, Firas Zghal, Slim Zarzissi, Raul Ioan Muntean, Mohamed Amine Bouzid

**Affiliations:** 1 Research Laboratory Education, Motricité, Sport et Santé, EM2S, LR19JS01, High Institute of Sport and Physical Education, University of Sfax, Sfax 3000, Tunisia; fekihnadia6@gmail.com (N.F.); amalmachfer@gmail.com (A.M.); zarzissislim@gmail.com (S.Z.); bouzid.mohamed-amine@hotmail.fr (M.A.B.); 2 Physical Education of Sports Teaching Department, Faculty of Sports Sciences, Atatürk University, Erzurum 25240, Türkiye; 3 Complexité Innovation Activités Motrices et Sportives (CIAMS), Université Paris-Saclay, 91400 Orsay, France; zghal.firas2012@yahoo.fr; 4 Department of Physical Education and Sport, Faculty of Law and Social Sciences, University “1 Decembrie 1918” of Alba Iulia, 510009 Alba Iulia, Romania

**Keywords:** neuromuscular fatigue, type 1 diabetes, critical threshold

## Abstract

**Background:** Type 1 diabetes (T1D) is associated with metabolic and neuromuscular impairments that may influence fatigue mechanisms and limit exercise tolerance. Although previous investigations have characterized muscle performance in T1D, the peripheral fatigue threshold, defined as the maximal sustainable level of peripheral fatigue, remains poorly understood in this population. This study aimed to compare the amplitude of the maximal peripheral fatigue threshold between individuals with T1D and healthy controls to elucidate the effects of T1D on neuromuscular function. **Methods:** Twenty-two participants (11 with T1D and 11 healthy controls) completed two randomized experimental sessions. In each session, 60 quadriceps maximal voluntary contractions (MVCs) were completed, performed for 3 s with 2 s of rest between contractions. One session was conducted under a non-fatigued control condition (CTRL), and the other followed a fatiguing neuromuscular electrical stimulation (FNMES) protocol. Central and peripheral fatigue were evaluated from the pre- to post-exercise changes in potentiated twitch force (ΔPtw) and voluntary activation (ΔVA), respectively. Critical torque (CT) was calculated as the average torque produced during the last 12 contractions, whereas the curvature constant of the torque–duration relationship (W′) was quantified as the area above CT. **Results:** Although both groups exhibited a decline in pre-exercise Ptw following the FNMES condition, no significant within-group differences in ΔPtw were observed between sessions (T1D: *p* = 0.34; controls: *p* = 0.23). Nevertheless, the extent of peripheral fatigue was significantly lower in participants with T1D than in controls (ΔPtw = −38 ± 11% vs. −52 ± 17%; *p* < 0.05). Additionally, W′ values were reduced by 24% in the T1D group relative to controls during the CTRL condition (*p* = 0.02), and CT was significantly lower in T1D participants (262 ± 49 N) compared to controls (353 ± 71 N; *p* < 0.01). A significant positive correlation was observed between ΔPtw and W′ across groups (*r*^2^ = 0.62, *p* < 0.001), suggesting a mechanistic link between peripheral fatigue tolerance and work capacity. **Conclusions:** The present results indicate that, although individuals with T1D retain the capacity to develop peripheral fatigue, their fatigue threshold and critical torque are markedly attenuated relative to those of healthy individuals. This reduction reflects impaired neuromuscular efficiency and diminished tolerance to sustained contractile activity. The strong relationship between peripheral fatigue and work capacity underscores the contribution of peripheral mechanisms to exercise intolerance in T1D. These results enhance current understanding of fatigue physiology in diabetes and emphasize the need for tailored exercise and rehabilitation strategies to improve fatigue resistance and functional performance in this population.

## 1. Introduction

Type 1 diabetes (T1D) is an autoimmune disorder marked by complete insulin deficiency. resulting from the loss of pancreatic β-cells [1]. Even with insulin therapy, patients with T1D often exhibit reduced exercise capacity and neuromuscular fatigue compared with healthy subjects, which can adversely affect their activities of daily living and work productivity [2].

Muscle fatigue is defined as a short-term reduction in a muscle’s capacity to produce the force or power needed during exercise [3]. This decline can stem from both central and peripheral mechanisms. Central fatigue involves a reduced capacity of the central nervous system to activate the working muscles [4] fully. In contrast, peripheral fatigue arises from biochemical alterations within the muscle tissue itself that impair its responsiveness to neural stimulation [5].

It is essential to mention that T1D can impair neuromuscular function by lowering motor unit firing frequency [6] and can also reduce maximal muscle force [7], particularly in the lower limbs [8], as well as a reduction in muscle mass [9]. In addition, some studies have reported a shorter time to exhaustion in patients with T1D [6,7,8,9,10]. In this way, Almeida et al. (2008) [6] reported that individuals with T1D exhibited a shorter time to failure (−45%) of the knee extensor muscles during submaximal isometric contraction and tended to exhibit lower fatigue levels than healthy control subjects. These data suggest that patients with type 1 diabetes are more fatigue-resistant during exercise than their healthy counterparts.

One hypothesis is that peripheral fatigue is centrally regulated so that exercise does not surpass a task-specific, individual critical threshold [11]. This regulation is mediated by sensory feedback from group III and IV muscle afferents to the central nervous system, which, in turn, reduces motor neuron output, thereby modulating the extent of peripheral fatigue during exertion [12,13,14,15]. This protective mechanism has been consistently demonstrated across different exercise modalities in athletes [16,17,18,19,20,21], older adults [22], and individuals with T1D [23]. While existing evidence confirms the preservation of this fatigue threshold mechanism in T1D [23], no studies have yet directly compared its magnitude to that in healthy individuals. Such comparisons are essential for understanding how neuromuscular fatigue regulation may differ in this population. Interestingly, data from the literature suggest that the effects of T1D on III/IV muscle afferents evolve over the course of the disease, leading not only to neuropathy but also to a decrease in sympathetic reflex activity [24], thereby altering III/IV muscle afferents.

Therefore, the objective of the present study was to determine the maximal task-specific peripheral fatigue that can be sustained, referred to as the critical threshold, in healthy and T1D participants. In addition, based on parameters derived from the force-duration relationship, we aimed to examine the relationship between the peripheral fatigue threshold and exercise capacity. Critical torque (CT) is mathematically defined as the asymptote of the hyperbolic force–duration relationship, while W′ is quantified as the area above CT [25]. In this way, several studies have shown that peripheral fatigue during exercise is correlated with W’ [23,24,25,26]. In addition, Broxterman et al. (2015) [27] reported that inducing greater peripheral fatigue by restricting blood flow during handgrip exercise was accompanied by a larger W’, indicating a potential mechanical relationship between these variables.

Our hypothesis in this study is that (i) people with T1D would exhibit a lower peripheral fatigue threshold compared with healthy participants, potentially contributing to their diminished exercise capacity, and (ii) that the impairment in exercise capacity would be particularly related to a reduced W’.

## 2. Materials and Methods

### 2.1. Participants

Twenty individuals with T1D were identified as eligible based on information from their medical records and recruited from the Endocrinology Department of Hedi Chaker University Hospital (Sfax, Tunisia). To be included in the study, they were required to be between 18 and 35 years old, have a confirmed T1D diagnosis for at least 3 years, and maintain a glycosylated hemoglobin (HbA1c) level below 10% during the 6 months preceding enrollment. Six individuals were excluded after screening due to fulfillment of the exclusion criteria: diabetes-related complications that could interfere with exercise (e.g., diabetic foot, proliferative retinopathy, or severe peripheral neuropathy), uncontrolled hypertension, a recent history of diabetic ketoacidosis (DKA) or severe hypoglycemic episodes within the past three months, or current use of lipid-lowering medications.

**Figure 1:**
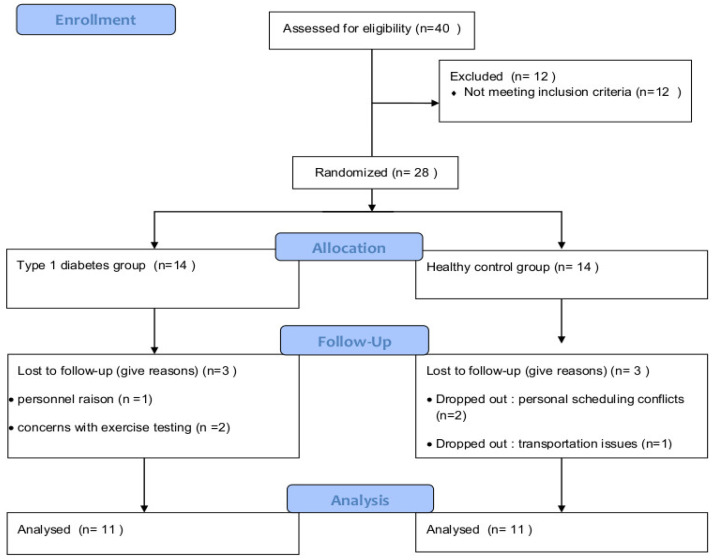
Consort Flow Diagram.

A control group (CG) (N = 11) was carefully selected to match the T1D group closely. Participants were selected from a pool of 60 candidates, primarily friends or acquaintances of patients with T1D. Each control subject was individually matched to a T1D participant using predefined criteria: same gender, age within ±5 years, and body mass index (BMI) within ±4 kg/m^2^ (Table 1). Our study included 14 T1D patients; however, three were excluded due to concerns about exercise testing or personal reasons. Thus, 11 participants with T1D were included in the data analysis (Figure 1). All participants in both groups were male to reduce gender-related variability in neuromuscular performance. Although participants reported recreational physical activity, no standardized physical activity data (e.g., IPAQ) were collected. Physical activity was not used as a matching criterion.

The study received approval from the Regional Research Ethics Committee (CPP SUD No. 0427; registration date: 15 January 2024) and was registered with the Pan African Clinical Trial Registry (PACTR202206634181851). All procedures complied with the ethical standards of the Declaration of Helsinki.

Sample size was estimated a priori using the approach described by Beck et al. (2013) [28] and G*Power software (version 3.1.9.4). The type I error rate was set at α = 0.05 and statistical power at 1 − β = 0.85. Guided by the findings of Almeida et al. (2008) [6] and consensus among the authors, an expected effect size of 0.8 was assumed. Accordingly, a minimum of 10 participants per group was required to detect a between-group difference in maximal voluntary force using a two-way repeated-measures ANOVA.

### 2.2. Study Design

#### 2.2.1. Protocol

Participants visited the laboratory on three occasions: one familiarization session and two subsequent sessions during which the experimental fatiguing exercise protocols were performed.

#### 2.2.2. Familiarization Session

To ensure consistent performance during the experimental trials, participants completed a familiarization session four days prior. During this session, they were introduced to all testing procedures and practiced maximal-effort testing with an isometric dynamometer. Upon arrival at the laboratory, anthropometric data were collected, and participants were evaluated for leg dominance and physical activity levels.

#### 2.2.3. Experimental Session

**Figure 2:**
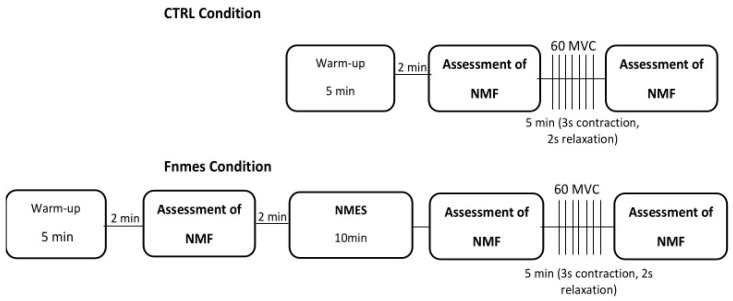
Schematic overview of the experimental design. Participants completed two sessions in random order: a control session (CTRL) and a session performed with pre-existing quadriceps fatigue induced by neuromuscular electrical stimulation (FNMES). Neuromuscular function (NMF) was assessed using isometric maximal voluntary contractions (IMVCs) with superimposed and subsequent peripheral nerve stimulation (PNS). Assessments were performed before and after the fatiguing exercise in both sessions and additionally after NMES in the FNMES condition. Abbreviations: NMES, neuromuscular electrical stimulation; NMF, neuromuscular function; IMVC/MVC, maximal voluntary contraction; PNS, peripheral nerve stimulation; CTRL, control session; FNMES, pre-existing quadriceps fatigue.

Participants completed two experimental sessions in a randomized order, separated by 3 to 7 days. Each session started with a standardized warm-up comprising several submaximal knee extensor contractions. (Figure 2). After the warm-up, participants completed 60 maximal voluntary isometric contractions (MVCs) within a 5 min period, with each contraction lasting 3 s followed by 2 s of rest, in accordance with the protocol described by Burnley (2009) [29]. One session (CTRL) was conducted under non-fatigued conditions, whereas the other (FNMES) was performed after inducing quadriceps fatigue via neuromuscular electrical stimulation.

Neuromuscular function was evaluated immediately before and after the exercise task in each session. In the FNMES condition, an additional assessment was conducted 1 min after electrical stimulation to quantify fatigue induced before exercise. To ensure consistency and optimize performance, an audio cue marked the beginning and end of each contraction, and participants received real-time visual feedback on a computer monitor. For each individual, the two sessions were scheduled at the same time of day and conducted under standardized ambient conditions (25 ± 1 °C).

#### 2.2.4. Neuromuscular Electrical Stimulation–Related Quadriceps Fatigue

During the FNMES session, quadriceps fatigue was elicited via neuromuscular electrical stimulation (NMES) prior to the voluntary exercise task, thereby minimizing the contribution of voluntary neural drive [20]. Stimulation electrodes were positioned over the motor points of the vastus medialis (VM) and vastus lateralis (VL), with the anode positioned just below the femoral triangle. A biphasic rectangular current (pulse duration: 400 μs) was delivered simultaneously to both muscles using a commercial stimulator (Genesy 1200 Pro, GLOBUS Italia SRL, Codognè, Italy). Stimulation was applied at 50 Hz for 7 s every 14 s (50% duty cycle).

Throughout the 10 min protocol, stimulation intensity was progressively increased to the maximum tolerable level for each participant. Since NMES tends to activate muscle fibers near the electrode surface and does not recruit motor units uniformly [30], maximizing the intensity was essential to achieve substantial fatigue. The effectiveness of the protocol was confirmed by a significant 10% decline in both maximal voluntary contraction (MVC) force and potentiated twitch (Ptw) measured immediately after the stimulation session.

#### 2.2.5. Data Acquisition and Analysis

##### Evaluation of Quadriceps Neuromuscular Function

Participants were seated on an isometric dynamometer (Good Strength, Metitur, Finland) fitted with a strain gauge. A cuff was attached to the dominant leg and secured with a rigid, non-elastic Velcro strap approximately 2 cm proximal to the lateral malleolus, allowing continuous measurement of quadriceps force. During all assessments, the knee joint was maintained at 90° of flexion relative to full extension.

Femoral nerve stimulation was delivered using a constant-current stimulator (Digitimer Limited, Hertfordshire, UK), providing single 1-ms square-wave pulses at a maximal output of 400 V. The cathode (10 mm Ag–AgCl self-adhesive electrode) was positioned over the femoral triangle, and the anode (10 × 5 cm; Compex Medical SA, Ecublens, Switzerland) was placed midway between the iliac crest and the greater trochanter.

Prior to testing, stimulation intensity was determined by progressively increasing current in 5-mA increments until both peak twitch force and the maximal compound muscle action potential (Mmax) reached a clear plateau in the vastus lateralis (VL), vastus medialis (VM), and rectus femoris (RF). To ensure supramaximal activation and mitigate potential confounding factors, such as axonal hyperpolarization [31], the stimulation current was set to 150% of the plateau value for all subsequent assessments.

Each maximal voluntary contraction (MVC) was accompanied by two nerve stimulations: one delivered during the MVC (superimposed twitch) and the second, three seconds afterward, to elicit a potentiated twitch (Ptw). This delay enabled mechanical potentiation, thereby enhancing measurement reliability relative to non-potentiated responses [32].

Peripheral fatigue was quantified as the change in Ptw between baseline and post-exercise or post-NMES (ΔPtw). Overall fatigue, encompassing both central and peripheral components, was quantified as the reduction in MVC from baseline to post-intervention (ΔMVC). Central fatigue was evaluated through voluntary activation (VA), calculated using the ratio of the superimposed twitch to the potentiated twitch, as initially described by Merton (1954) [33]:

Voluntary activation % = [(1 − Superimposed twitch)/(Potentiated twitch)] × 100

##### Electromyography

Electromyographic (EMG) activity was recorded using bipolar silver chloride surface electrodes (Dormo Electrodes, SX-50 ECG, Telic Group, Barcelona, Spain). They were positioned longitudinally along the muscle belly in accordance with the SENIAM (Surface EMG for the Non-Invasive Assessment of Muscles) guidelines, using a 20 mm inter-electrode distance, with the reference electrode positioned on the patella. Prior to electrode placement, the skin was shaved, gently abraded with emery paper, and cleaned with alcohol to ensure low skin–electrode impedance (Z < 5 kΩ). To improve consistency between sessions, electrode locations were marked using indelible ink.

EMG signals were amplified using a multichannel system (Octal Bio Amp ML 138, ADInstruments, Bella Vista, Australia) with a bandwidth of 10 Hz–1 kHz and a common-mode rejection ratio > 96 dB. Force and EMG signals were acquired simultaneously with a PowerLab 16SP system (ADInstruments, Australia) and recorded in LabChart 7.0 at a sampling rate of 2 kHz.

For each potentiated twitch (Ptw), the peak-to-peak amplitude of the compound muscle action potential (M-wave; Mmax) was determined. During the 5 min MVC protocol, EMG activity of the rectus femoris (RF), vastus lateralis (VL), and vastus medialis (VM) was quantified using root mean square (RMS) values. RMS was calculated over a fixed 0.5-s window immediately before the superimposed-stimulation artefact, with each EMG burst identified by visual inspection to ensure accurate window placement and consistency across all MVCs

##### Determination of CT and W’

The critical torque (CT) during the 60-MVC protocol was calculated as the average force over the final 12 contractions (the last 60 s of the task). The W’ parameter was calculated as the area under the force-time curve that exceeded the CT threshold, as previously described [30].

### 2.3. Statistical Analyses

All data are reported as mean ± standard deviation (SD). Statistical analyses were conducted using Statistica for Windows (v12.0). Data distribution and homogeneity of variance were assessed with the Shapiro–Wilk and Levene’s tests, respectively. Repeated-measures ANOVA (condition × group × time) was used to examine differences in force output, RMS/Mmax, and fatigue-related variables (MVC, Ptw, VA, and Mmax). For CT and W’, between-condition and between-group differences were evaluated using a two-way ANOVA (condition × group). Where a significant interaction was observed, pairwise comparisons were performed using Fisher’s LSD post hoc procedure. Associations between Ptw and W’ were examined by calculating individual Pearson correlation coefficients (r).

## 3. Results

### 3.1. Baseline Neuromuscular Function

At rest, neuromuscular function (MVC, Ptw, NAV) was similar between the two conditions (CTRL and FNMES) in the T1D and control groups (Table 1).

Although a similar VA level between the two groups (*p* = 0.07), MVC and Ptw values were lower in the T1D group compared with the control group (MVC: ~517 ± 132 N vs. ~651 ± 111 N; Ptw: ~137 ± 19 vs. ~166 ± 23, respectively) (*p* < 0.05).

### 3.2. Effects of NMES on Quadriceps Fatigue

Following the electrostimulation protocol, we observed significant reductions in MVC (T1D: −7.12 ± 4.91% vs. controls: −11.15 ± 9.48%), Ptw (T1D: −9.66 ± 8.33% vs. controls: −14.36 ± 7.37%), and VA (0% vs. −2.1 ± 3.7%, *p* = 0.24) in both groups, indicating a marked level of quadriceps fatigue (Table 2 and Table 3). A significant decrease in Mmax was also detected. To account for potential NMES-induced alterations in membrane excitability, RMS values were normalized to the baseline maximal M-wave amplitude (or to the Mmax obtained immediately after NMES in the FNMES condition) [14].

### 3.3. 60-MVCs Protocol

#### 3.3.1. Exercise Performance

**Figure 3:**
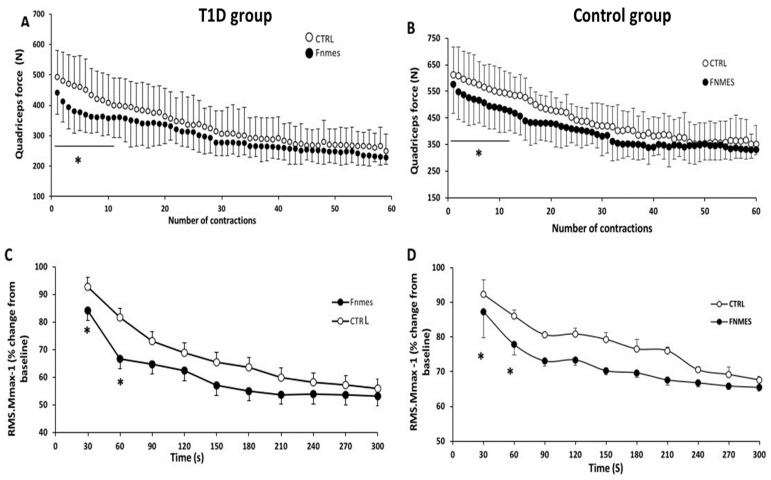
Maximal voluntary contraction (MVC; (**A**,**B**)) and quadriceps muscle activation (RMS/Mmax; (**C**,**D**)) throughout the 60-MVC protocol performed with pre-existing quadriceps fatigue (FNMES) or without pre-fatigue (CTRL) in the T1D and control groups. * *p* < 0.05 vs. CTRL (ANOVA). Abbreviations: FNMES, pre-existing fatigue induced by neuromuscular electrical stimulation; MVC, maximal voluntary contraction; RMS, root mean square.

Changes in MVC during exercise are shown in Figure 3A,B. Resting MVC values in the CTRL condition were similar to those recorded during the first MVC of exercise in both groups (control group, 632.89 ± 94.81 N vs. 628.23 ± 107.76 N, *p* = 0.77; T1D group, 502.11 ± 131.84 N vs. 493.14 ± 88.24 N, *p* = 0.74).

Statistical analysis showed a significant reduction in MVC during the first 12 MVC (14 ± 3% in the T1D group and 11 ± 2% in the control group) in FNMES compared with CTRL, as a result of the pre-existing quadriceps fatigue.

In the CTRL session, MVC development was reduced by 25 ± 1% in the T1D group compared with the control group (*p* = 0.049). Force output declined progressively from MVC 1 to MVC 45 in the control group and from MVC 1 to MVC 40 in the T1D group. Thereafter, values stabilized, remaining at a plateau in both groups until the final contraction.

#### 3.3.2. Electromyography

Figure 3C,D shows the evolution of the RMS.Mmax^−1^ values during exercise. RMS. The level of preexisting quadriceps fatigue modulated Mmax^−1^. During the first minute of exercise, RMS.Mmax^−1^ was lower in the FNMES condition compared with the CTRL (−14 ± 6% in the T1D group vs. −7 ± 3% in the control group). However, we noted no difference in RMSMax^−1^ values between the two conditions until the end of exercise in both.

#### 3.3.3. Parameters Associated with the Force-Duration Relationship

**Figure 4:**
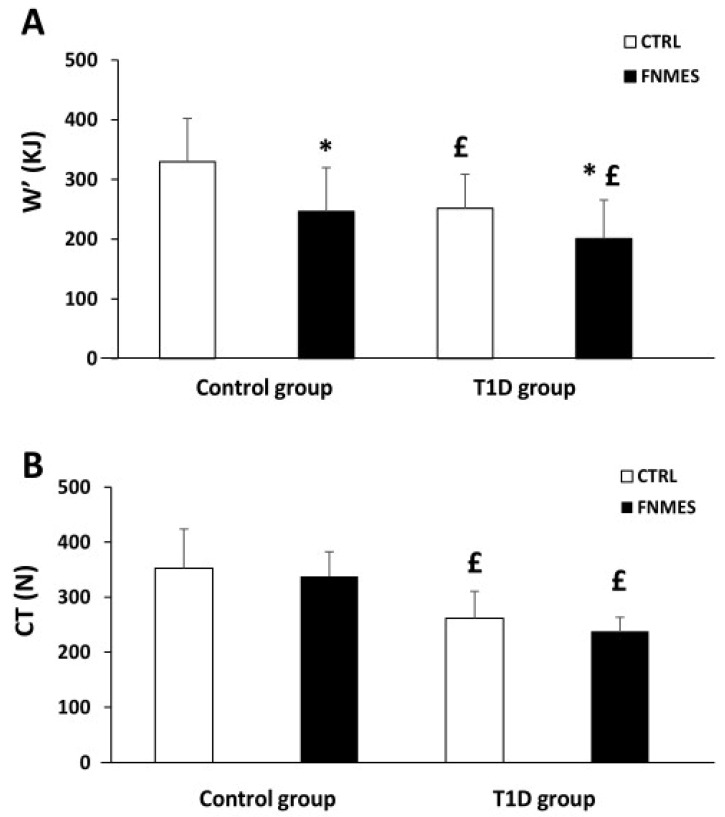
Work performed above critical force (W′) during exercise (**A**) and critical torque (CT) (**B**) calculated from the final 12 MVCs (**B**), under pre-existing quadriceps fatigue (FNMES) and control (CTRL) conditions, in participants with T1D and matched controls. Statistics (ANOVA): * *p* < 0.05 vs. CTRL; ^£^ *p* < 0.05 vs. healthy participants.

Our results showed that in the CTRL condition, W’ values in the T1D group were 24% lower than in the control group (*p* = 0.02). (Figure 4A). In addition, compared to the CTRL session, FNMES reduced W’ in both groups (*p* < 0.001). However, this reduction in W’ was similar in both groups (control: 25 ± 2% vs. T1D: 19 ± 3%, *p* = 0.58).

Moreover, our results showed that CT values were lower in the T1D group (262 ± 49 N) compared with the control group (353 ± 71 N) (*p* < 0.01). Furthermore, compared with the CTRL condition, FNMES did not alter CT in either group (Figure 4B).

### 3.4. Central and Peripheral Fatigue

Table 1 and Table 2 present neuromuscular fatigue parameters for both conditions and groups. Although FNMES significantly reduced Ptw prior to the exercise protocol, the change in Ptw (∆Ptw) did not differ between CTRL and FNMES in either the control group (*p* = 0.23) or the T1D group (*p* = 0.34). In contrast, the peripheral fatigue threshold was significantly greater in controls than in participants with T1D (∆Ptw = −52 ± 17% vs. −38 ± 11%, respectively; *p* < 0.05). Likewise, MVC and voluntary activation (VA) decreased following exercise, with no significant differences between the CTRL and FNMES conditions (Table 1). Finally, there was no significant difference in Mmax values either between the two groups or between the two conditions (Table 1) (*p* > 0.05).

**Figure 5:**
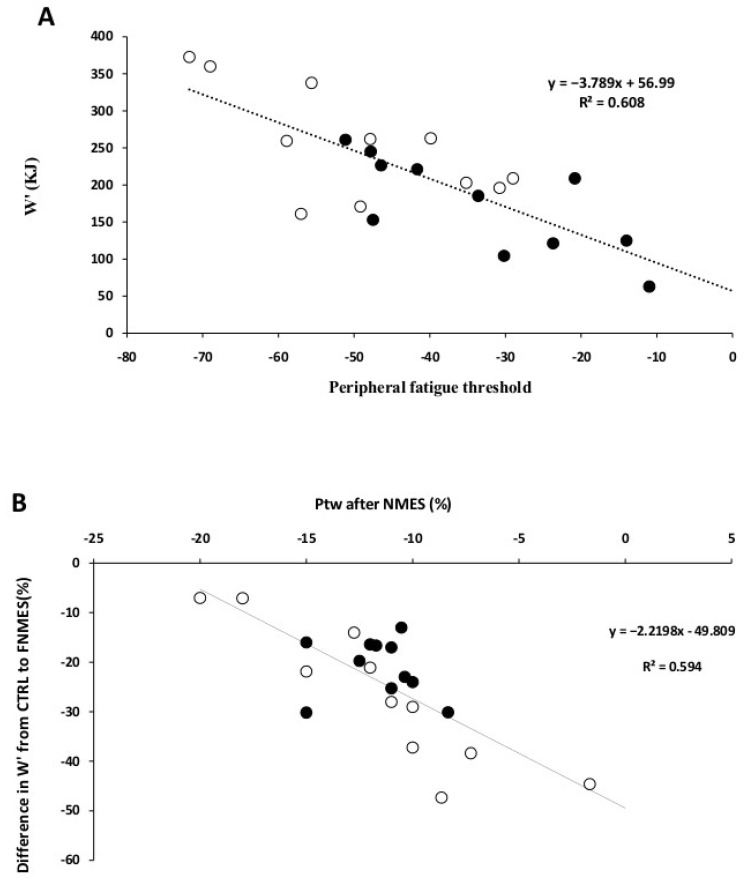
Correlations between W′ and the peripheral fatigue threshold (**A**), and between ΔW′ (CTRL to FNMES) and the level of NMES-induced peripheral fatigue (pre-fatigue) (**B**). Pearson correlation analysis. FNMES, pre-existing fatigue condition induced by neuromuscular electrical stimulation; NMES, neuromuscular electrical stimulation.

As illustrated in Figure 5A, the critical threshold level (ΔPtw) was significantly associated with W′ in both groups (r^2^ = 0.62, *p* < 0.001). Moreover, the change in W′ between the CTRL and FNMES conditions was correlated with the magnitude of NMES-induced pre-fatigue (r^2^ = 0.59, *p* < 0.001) (Figure 5B).

## 4. Discussion

The present study aimed to investigate whether individuals with T1D exhibit alterations in the maximal level of peripheral fatigue, commonly referred to as the “critical threshold”, compared with healthy controls. To address this, we employed anNMES protocol to induce significant quadriceps fatigue without engaging central motor drive (i.e., in the absence of voluntary feedforward activation) before performing a 60-repetition maximal voluntary contraction (MVC) task.

This method is appropriate for detecting the individual-specific, task-specific fatigue threshold set by inhibitory afferent feedback, thereby allowing the same degree of peripheral fatigue to be achieved between FNMES and CTRL despite substantial pre-existing fatigue at a given time (FNMES). In line with our hypothesis, the peripheral fatigue threshold was lower in the T1D group than in the healthy group, at least in part explaining the reduced exercise capacity in the T1D group. Moreover, the lower exercise capacity observed in participants with T1D was accompanied by a reduction in W′. In addition, a strong association was identified between the individual peripheral fatigue threshold and W′ in both groups. Collectively, these findings indicate that reduced fatigue tolerance in T1D contributes substantially to their impaired exercise capacity.

### 4.1. Exercise Capacity and Peripheral Fatigue Tolerance

The present findings revealed a positive association between the amount of peripheral fatigue tolerated during exercise and W′ (Figure 3A). Notably, when a pre-fatigue intervention lowered the level of peripheral fatigue prior to the exercise protocol, W′ decreased in a dose-dependent fashion, supporting a mechanical relationship between these two variables (Figure 4B). This result is in agreement with other research supporting the link between W’ and peripheral fatigue using various experimental procedures [22,23,26,27,28,29,30,31,32,33,34]. Our findings demonstrate that W’ is determined by the maximum tolerable level of peripheral fatigue during isometric exercise. Therefore, reduced exercise capacity observed in T1D could be explained, in part, by the considerable reduction in this peripheral fatigue threshold.

Moreover, CT, expressed in absolute units (N), was comparable across conditions but was lower in the T1D group than in the healthy group. Previous research has shown that higher muscle activation and power output during short-duration efforts likely reflect a greater reliance on fatigue-susceptible fast-twitch motor units for force generation [35]. Consistent with this, individuals with T1D have been reported to exhibit a higher proportion of type II (fast-twitch) muscle fibers [36,37]. These fibres typically have greater phosphocreatine stores, higher glycolytic enzyme activity, and a lower oxidative capacity than slow-twitch fibres. During high-intensity exercise, their rapid ATP turnover and phosphocreatine breakdown promote the accumulation of fatigue-related metabolites, including inorganic phosphate and adenosine diphosphate. These muscle fibers exhibit elevated levels of phosphocreatine [38], a reduced oxidative capacity relative to slow-twitch fibers, and greater activity of ATPase and glycolytic enzymes [39]. During high-intensity exercise, the rapid breakdown of ATP and phosphocreatine in these fibers results in the accumulation of fatigue-related metabolites, including inorganic phosphate and adenosine diphosphate, which contribute to the development of peripheral fatigue [5]. We therefore hypothesized that the difference in CT between TD1 and healthy participants was attributable to impaired progression of intramuscular phosphagen resynthesis and metabolite elimination in T1D. However, this interpretation cannot be confirmed, as muscle biopsies were not collected in the present study.

### 4.2. Mechanistic Basis of Reduced Fatigue Tolerance in Type 1 Diabetes

Several studies indicate that peripheral fatigue is regulated to remain within an individual, task-specific critical threshold during exercise [12,13,14,15,16,17,18,19,20,21,22]. Notably, recent evidence suggests that this constraint is also present in individuals with T1D [23]. In the present study, although the quadriceps were pre-fatigued in the FNMES condition using electrically evoked contractions, the magnitude of peripheral fatigue reached at the end of the 60-MVC protocol was comparable between CTRL and FNMES, in line with the literature cited above. By directly comparing individuals with T1D and healthy controls, the present study found that the peripheral fatigue threshold was lower in individuals with T1D than in healthy controls, providing new insights into neuromuscular function in T1D. Indeed, previous studies have demonstrated that afferent feedback from group III/IV muscles reduces motoneuronal output during fatiguing exercise, thereby helping to control the development of peripheral fatigue [12,13,14,15]. In the present study, a potential contributor to the reduced fatigue threshold could be altered group III/IV afferent feedback, which has been previously reported in diabetic animal models but remains unverified in human T1D populations. Ishizawa et al. (2020) [40] demonstrated that group IV afferents in T1D rats exhibit an exaggerated response to mechanical stimulation compared to healthy controls. Similar findings have been reported in cutaneous C-fibers, in which increased mechanical sensitivity has been observed in animal models of painful diabetic neuropathy [41,42]. Furthermore, spontaneous hyperactivity of group III and IV muscle afferents has been documented in diabetic animals even in the absence of external stimuli [40,41]. This aberrant activity may sensitize these afferents, making them more responsive to mechanical and chemical cues and potentially contributing to the exaggerated exercise pressor reflex commonly seen in individuals with T1D. On the other hand, in the present study, we did not directly alter afferent feedback; instead, we modified their activity via electrostimulation. Indeed, EMG data showed a greater reduction in RMS/Mmax^−1^ values in T1D than in healthy subjects during the last minute of exercise, supporting the idea that afferents III/IV are altered in T1D. Together, these data support the hypothesis that group III/IV muscle afferents are modified in patients with T1D.

Given that group III/IV muscle afferent feedback can directly modulate exercise performance [14,15,16,17,18,19,20,21,22,23,24,25,26,27,28,29,30,31,32,33,34,35,36,37,38,39,40,41,42,43], and considering the hypothesized impairment of this afferent pathway in individuals with T1D, a reduction in exercise capacity would be expected in this population. Supporting this rationale, attenuating group III/IV muscle afferent feedback in young adults during the same 60-MVC protocol used here resulted in ~7% greater performance during the first minute of exercise [43]. Moreover, the lower level of peripheral fatigue in the T1D group relative to healthy controls may reflect a greater reliance on, or a higher proportion of, fast-twitch fibers in T1D patients (speculative, as this was not directly measured). However, this hypothesis requires direct validation via muscle biopsy. Therefore, according to the results of this study, we can put forward two hypotheses: the first hypothesis is that T1D causes a rapid accumulation of metabolites in the muscle, which leads to rapid stimulation of the III and IV afferents to limit exercise, or that T1D affects the activity and sensitivity of afferents III and IV. These hypotheses remain unconfirmed, as no study has directly investigated muscle afferences III and VI in subjects with T1D.

Despite its strengths, this study has several limitations. First, it included a relatively small sample size (n = 11 per group), which, although sufficient for group comparisons, restricted our ability to stratify participants by HbA1c or disease duration. Additionally, all participants were male, limiting the applicability of our findings to females. Future research should recruit larger, sex-balanced cohorts and further investigate how glycemic control affects fatigue thresholds. Second, we recognize the lack of quantitative data on physical activity as a limitation. Variations in habitual activity may influence fatigue thresholds; therefore, future studies should incorporate validated activity assessments (questionnaires or wearable devices) to better control for this potential confounder factor.

## 5. Conclusions

The present work contributes a new mechanistic understanding of the factors underlying exercise-induced neuromuscular fatigue. and the regulatory mechanisms of peripheral fatigue in patients with T1D. As in healthy young individuals, peripheral fatigue in T1D is limited to a single threshold. However, the tolerable level of peripheral fatigue during isometric exercise was lower in individuals with T1D than in healthy controls. Managing peripheral fatigue to a critical threshold is advantageous, as it helps maintain locomotor muscle function during exercise in patients with T1D, who often experience peripheral neuropathy. Additionally, the study provides direct evidence of the relationship between peripheral fatigue and W’, thus offering indirect evidence of how this disease affects the action of group III/IV muscle afferents. Finally, the reduction in the maximum level of peripheral fatigue that can be achieved (i.e., fatigue thresholds) during isometric exercise contributes to the decreased exercise capacity observed in this population.

## Figures and Tables

**Table 1:** Participant characteristics.

	T1D	CTR	*p* Value
Total (Male)	11	11	
Age (years)	22.5 ± 2.5	24.1 ± 3.1	0.89
Fat mass (%)	15.2 ± 2.9	14.5 ± 1.7	0.78
Height (m)	1.7 ± 0.14	1.7 ± 0.19	0.95
Body mass (kg)	71.4 ± 5.1	74.4 ± 4.8	0.68
BMI (kg m^−2^)	22.4 ± 2.8	23.4 ± 2.2	0.79
HbA_1c_ (%)	8.9 ± 0.9	-	
Diabetes duration (years)	14.5 ± 8.2	-	
Total insulin dose per day (U kg^−1^)	0.7 ± 0.2	-	

**Table 2:** Neuromuscular function outcomes under pre-existing quadriceps fatigue (FNMES) and control (CTRL) conditions in individuals with type 1 diabetes (T1D) and healthy controls (CTR).

	Group	Condition	Pre	Post-NMES	Post
MVC	Control	CTRL	632.9 ± 94.8	-	468.4 ± 48.1 *
		FNMES	668.1 ± 127.6	591.9 ± 120.5 *	486.4 ± 60.9 *^#^
	T1D	CTRL	502.1 ± 131.9 ^£^	-	351 ± 97.9 *
		FNMES	532.6 ± 131.6 ^£^	493.6 ± 118.8	351.8 ± 75.2 *^#^
P_tw_ (N)	Control	CTRL	162.6 ± 23.1	-	79.2 ± 22.9 *
		FNMES	168.6 ± 23.3	144.6 ± 25.1 *	85.8 ± 27.2 *^#^
	T1D	CTRL	138.8 ± 21.3 ^£^		80.9 ± 29.3 *
		FNMES	134.3 ± 16.5 ^£^	121.7 ± 19.5	82.9 ± 16.7 *^#^
VA (%)	Control	CTRL	95.1 ± 1.5		89.8 ± 1.6 *
		FNMES	95 ± 2.5	93 ± 3.3	88.9 ± 4.1 *^#^
	T1D	CTRL	93 ± 3		86.5 ± 4.1 *
		FNMES	93.3 ± 2.5	93.3 ± 3	84.8 ± 5.3 *^#^
VL Mmax (mv)	Control	CTRL	7.2 ± 3.6		4.6 ± 1.8 *
		FNMES	7.1 ± 2.3	5.4 ± 3.5	4.8 ± 2.7
	T1D	CTRL	5.2 ± 2		3.5 ± 1.7 *
		FNMES	5.3 ± 2.8 ^£^	5 ± 2.9	3.4 ± 2.4 *^#^
VM Mmax (mv)	Control	CTRL	7.7 ± 3.5		5.2 ± 3.1 *
		FNMES	7 ± 2.6	5.5 ± 3.1	4.9 ± 2.2 *
	T1D	CTRL	6.1 ± 2.1		4.4 ± 2.2
		FNMES	5.7 ± 3.3 ^£^	5 ± 3.4	4.1 ± 2.3 *
RF Mmax (mv)	Control	CTRL	5.4 ± 3.4		4.2 ± 3.3
		FNMES	5.3 ± 1.7	4.5 ± 1.8	4.1 ± 1.6 *
	T1D	CTRL	5.1 ± 2.4		3.9 ± 1.7
		FNMES	5.1 ± 2.2	4.8 ± 2.1	3.8 ± 1.7 *^#^

Abbreviations: MVC, maximal isometric voluntary contraction; NMES, neuromuscular electrical stimulation; *P_tw_* quadriceps potentiated twitch force; VA, voluntary activation of quadriceps motor units; M-wave, maximal muscle action potential; *VL*, vastus lateralis; *VM*, vastus medialis; *RF*, rectus femoris. * *p* < 0.05 vs. baseline values. ^#^ *p* < 0.05 vs. POST-NMES values. ^£^ *p* < 0.05 vs. control group.

**Table 3:** Main effects by ANOVA regarding neuromuscular parameters.

Variable	Main Effect: Group	Main Effect: Condition	Main Effect: Time	Interaction
MVC	F_(1,10)_ = 13.5, *p* = 0.05, ηp^2^ = 0.41	F_(1,10)_ = 5.42, *p* = 0.35, ηp^2^ = 0.08	F_(1,10)_ = 18.45, *p* < 0.01, ηp^2^ = 0.68	F_(2,20)_ = 6.7, *p* = 0.24, ηp^2^ = 0.11
Ptw	F_(1,10)_ = 20.4, *p* < 0.01, ηp^2^ = 0.65	F_(1,10)_ = 6.55, *p* = 0.13, ηp^2^ = 0.19	F_(1,10)_ = 31.1, *p* < 0.01, ηp^2^ = 0.75	F_(2,20)_ = 8.1, *p* = 0.2, ηp^2^ = 0.2
VA	F_(1,10)_ = 23.8, *p* < 0.01, ηp^2^ = 0.70	F_(1,10)_ = 7.88, *p* = 0.27, ηp^2^ = 0.08	F_(1,10)_ = 16.5, *p* < 0.01, ηp^2^ = 0.57	F_(2,20)_ = 9.1, *p* =0.14, ηp^2^ = 0.19
VL Mmax	F_(1,10)_ = 19.4, *p* =0.02, ηp^2^ = 0.64	F_(1,10)_ = 3.23, *p* = 0.32, ηp^2^ = 0.03	F_(1,10)_ = 21.1, *p* < 0.01, ηp^2^ = 0.74	F_(2,20)_ = 4.3, *p* =0.25, ηp^2^ = 0.08
VM Mmax	F_(1,10)_ = 4.21, *p* = 0.32, ηp^2^ = 0.03	F_(1,10)_ = 2.5, *p* = 0.76, ηp^2^ = 0.01	F_(1,10)_ = 28.15, *p* = 0.002, ηp^2^ = 0.78	F_(2,20)_ = 33, *p* = 0.51, ηp^2^ = 0.02
RF Mmax	F_(1,10)_ = 2.2, *p* = 0.82, ηp^2^ = 0.02	F_(1,10)_ = 5.5, *p* = 0.76, ηp^2^ = 0.01	F_(1,10)_ = 6.20, *p* = 0.09, ηp^2^ = 0.31	F_(2,20)_ = 5.3, *p* =0.49, ηp^2^ = 0.09

## Data Availability

Data may be made available upon reasonable request to the corresponding author, subject to approval by the Regional Research Committee for Medical and Health Research Ethics and the local Data Protection Officer.

## Trial Registration

Pan African Clinical Trial Registry Identifier: PACTR202510823123375. Registered trial name: Neuromuscular fatigue regulation and metabolic disorders during exercise in patients with type 1 diabetes.
